# Encapsulation and Functional Activity of *Lactobacillus reuteri* Strains: Advances, Challenges, and Perspectives

**DOI:** 10.1111/1541-4337.70412

**Published:** 2026-02-24

**Authors:** León‐Espinosa Erika Berenice, Barrios‐Francisco Rigoberto, Colin‐Molina Abraham, Martínez‐Palma Nikte Yoliztli, Rentería‐Ortega Minerva

**Affiliations:** ^1^ Tecnológico Nacional de México/Instituto Tecnológico Superior de San Felipe del Progreso San Felipe del Progreso México; ^2^ División de Procesos Industriales Universidad Tecnológica de Tecámac Sierra Hermosa Tecámac México

**Keywords:** biopolymer matrices, functional foods, *Lactobacillus reuteri*, probiotic encapsulation

## Abstract

Over the past decade, probiotics have gone from been special health supplements to widely incorporated components in many foods and nutrition products. This has led to more careful checks of their safety, viability, and functional performance under realistic processing and consumption conditions. Within lactic acid bacteria, *Limosilactobacillus reuteri* (formerly *Lactobacillus reuteri*) has been extensively investigated for its immunomodulatory, antimicrobial, anti‐inflammatory, antioxidant, and metabolic properties as demonstrated across multiple in vivo and in vitro experimental models. Despite these functional attributes, its viability during food processing and gastrointestinal transit remains strongly influenced by the strain and the characteristics of the delivery matrix; as a result, encapsulation is an essential strategy to preserve cellular integrity and functionality. The present review examines the functional properties of *Limosilactobacillus reuteri* (*L. reuteri* or *LR*) strains alongside the technological approaches used for their encapsulation. Current encapsulation approaches, including ionic gelation, extrusion, electrospray, and spray drying combined with biopolymers to improve encapsulation efficiency and survival of strains such as DSM 17938 and DSM 20016, are discussed with emphasis on their applicability to probiotic delivery and the formulation of functional foods with potential to improve gastrointestinal health, modulate inflammation, and enhance metabolic functions. In addition to strain‐specific functional activities, this review examines how coating material, processing method, and strain affect release kinetics, functional activities, and viability. However, data on the stability of encapsulated *L. reuteri* during industrial processing, storage, and health claim validation under regulatory frameworks remain limited. Future research should address these challenges to support the use of *L*. *reuteri* in functional foods and therapeutic products.

## 1 Introduction

The growing demand of foods that provide health benefits has accelerated the development of formulations fortified with probiotic microorganisms (Damián et al. 2022; Das et al. 2016). Within this group, strains of *Limosilactobacillus reuteri* have garnered sufficient interest due to their specific functions, such as immunomodulation, production of antimicrobial metabolites, for example, reuterin, reduction of total cholesterol and triglycerides, inhibition of *Helicobacter pylori*, and their ability to colonize different host niches.

Despite its multiple benefits, the application of *L. reuteri* in food matrices remains complex, as the microorganism is sensitive to food processing conditions, for example, heating, dehydration, and oxygen exposure, but also to gastrointestinal challenges such as pH, bile salts, and digestive enzymes (Algaithi et al. 2022). These limitations tend to reduce the viability and functional activity of *L. reuteri*, compromising its efficacy when administered through food matrices.

In this sense, encapsulation has emerged as a key strategy to improve the stability, survival, and targeted release of *L. reuteri*, both in food and throughout the gastrointestinal tract (Anal and Singh 2007; Cook et al. 2012). Several studies have explored the use of different coating materials, for example, alginate, pectin, proteins, starch, and polysaccharides, as well as different encapsulation methods such as extrusion, emulsification, ionic gelation, electrospraying, coacervation, and spray drying, with the aim of improving probiotic functionality (Algaithi et al. 2022; Rokka and Rantamaki 2010; Nazzarro et al. 2012).

However, despite the increasing number of experimental studies, literature still presents several gaps in this area. Nowadays, most research focuses on the individual strains, isolated functional activity, or specific encapsulation approaches, without adequately integrating how strain‐dependent characteristics align with encapsulation design or how these interactions may be translated into technological performance in real word food systems (Gbassi and Vandamme 2012; Rokka and Rantamaki 2010). According to the above, this review aims to integrate current knowledge on the functional properties of *L. reuteri* with the role of encapsulation strategies in preserving its viability during processing and gastrointestinal transit, considering how strain characteristics, encapsulation methods and coating materials impact functional activity and technological viability in food matrices

### 1.1 Background on Probiotics and Viability in Food Systems

Globally, there has been an increasing interest in foods that provide health‐promoting benefits beyond basic nutrition, along with a growing demand for transparency regarding ingredients, additives, and the inclusion of functional components such as probiotic microorganisms. In this context, various validation approaches such as standardizer in vitro gastric digestion models have been developed to assess digestibility, bioaccessibility, stability, and release of nutrients and bioactive compounds. These methodologies have been extensively used to assess the survival and stability of probiotic microorganisms under simulated gastrointestinal conditions, thereby providing valuable insights into the challenges associated with their incorporation into food matrices (Brodkorb et al. 2019; Minekus et al. 2014).

A fundamental requirement for probiotics to exert their intended physiological effects is their capacity to reach the intestine in sufficient numbers while maintaining viability during food processing, storage, and gastrointestinal transit (FAO/WHO 2002; Tripathi and Giri 2014). However, exposure to heat, mechanical stress, oxygen, moisture, acidity, and bile salts can substantially decrease cell survival. Consequently, preserving probiotic viability remains one of the major technological challenges in the development of functional foods (Tripathi and Giri 2014).

### 1.2 State of the Art on Encapsulated *Limosilactobacillus reuteri*

Among the assorted genera used in probiotic applications, it can be found *Lactobacillus* and *Bifidobacterium*; however, *L. reuteri* stands out due to its unique biological and functional properties (Algaithi et al. 2022). Particularly, *LR* is an intestinal symbiont that can colonize gastrointestinal tract in various mammals, including humans. It is tolerant to gastric acidity and bile, but also produces many of the essential compounds responsible for gut health (Liu et al. 2016; Yu et al. 2023).

Nevertheless, in recent decades, there has been a considerable decrease in the presence of *L. reuteri* in humans, which have been correlated with an increase in the incidence of inflammatory diseases (e.g., ulcerative colitis, Crohn's disease, and indeterminate colitis). For the above, research works attending the development of functional foods, particularly those containing probiotic strains, are imperative for addressing some of the public health issues mentioned.

Over the past decade, research on *L. reuteri* encapsulation has expanded substantially, motivated by the need to enhance cell survival during food processing, storage, and gastrointestinal transit (Anal and Singh 2007; De Prisco and Mauriello 2016; Tripathi and Giri 2014). Various encapsulation materials, such as alginate, pectin, starch derivatives, chitosan, whey proteins, mucilage, inulin, and multilayer coatings, have been investigated alongside methods including extrusion, emulsification, ionic gelation, spray drying, and electrospraying (Anal and Singh 2007; De Prisco and Mauriello 2016). While these approaches have consistently demonstrated improvements in viability; the extent of protection is highly dependent on the chemical composition of the wall material, capsule structure, drying conditions, and strain‐specific physiological characteristics.

A key challenge in current research is the strain‐dependent response of *L. reuteri*. Strains such as DSM 17938, DSM 20016, CRL 1324, ATCC 55730, and others show significant differences in acid–bile tolerance, adhesion, exopolysaccharide (EPS) production, reuterin synthesis, and dehydration sensitivity (Britton et al. 2014; Mu et al. 2018a; Walter et al. 2011). Despite these differences, many studies assess encapsulation effects on specific functions without directly linking these outcomes to material selection or capsule architecture. This limits the ability to establish structure–function relationships or identify optimal material–strain combinations (De Prisco and Mauriello 2016; Tripathi and Giri 2014).

Although previous reviews have explored either the probiotic activity or encapsulation strategies of *L. reuteri*, only a few numbers of they have provided an integrated analysis that connects specific strains, coating materials, encapsulation methods, and their technological and health‐related implications. Most existing reviews either focus narrowly on clinical aspects or provide general overviews of probiotic encapsulation without discriminating against bacterial species or materials. Table 1 addresses gaps in the general overview by providing an integrative comparison of representative encapsulation strategies for *L. reuteri*. It organizes studies by wall material and processing technique, and highlights encapsulation efficiency (EE), postprocessing viability, gastrointestinal survival, and functional performance.

**TABLE 1:** Integrative comparison of encapsulation strategies for *Limosilactobacillus reuteri*: Materials, processing methods, and quantitative functional outcomes.

Strain	Wall material(s)	Encapsulation technique (key conditions)	Postprocessing viability/EE	Storage stability	GI survival/release behavior	Functional outcome	References
*Limosilactobacillus reuteri* HR7	Metal–phenolic network (tannic acid + Fe^3^^+^) + HA‐SH nanoencapsulation	Single‐cell nanoencapsulation (metal coordination + HA‐SH coating)	Maintains ∼6 log CFU/mL under simulated GI, heat, oxidative stress	Enhanced viability under storage and stress relative to free cells	Improved survival under simulated GI conditions	High tolerance to adverse environments; preserved viability and growth	Yang et al. (2025)
*Limosilactobacillus reuteri* HR7	Metal–phenolic network (tannic acid + Fe^3^^+^) + HA‐SH nanoencapsulation |	Single‐cell nanoencapsulation (metal coordination with Fe^3^^+^ and tannic acid followed by HA‐SH coating)	Maintains ∼6 log CFU/mL under simulated gastrointestinal, thermal and oxidative stress conditions	Enhanced viability under storage and stress conditions compared to free cells	Improved survival under simulated gastrointestinal conditions	High tolerance to adverse environments; preserved viability and growth performance	Yang et al. (2025)
*Lactobacillus reuteri* (PTCC‐1655)	Sodium alginate (1% w/v) as first layer + chitosan (1% w/v) as second layer on MCC carrier (double‐coated microcapsules)	Double coating by Wurster fluidized‐bed process (first layer: sodium alginate 0.5%–1.5% w/v; second layer: chitosan 0.5%–1.5% w/v or arabic gum 1.5%–6% w/v; optimized at alginate 1% + chitosan 1%)	Relative survival after simulated gastric conditions (pH 2, 1 h): 11.6% for double‐coated alginate–chitosan microcapsules; significantly higher than monolayer systems	Not evaluated during long‐term storage; focus on thermal and acid resistance	Improved resistance under simulated gastric conditions and heat treatment (80°C for 15 min: 7.31% survival; 30 min: 0.63%) compared to single‐layer microcapsules	Double‐layer architecture enhances protection against low pH and high temperature, demonstrating structure–function relationship between multilayer coating and probiotic survival	Zaghari et al. (2020)
*Lactobacillus reuteri* B2	Sodium alginate (Na‐alg) and sodium alginate–starch maleate (Na‐alg + SM) calcium‐crosslinked beads	Microencapsulation by extrusion–gelation: Na‐alg or Na‐alg + SM solutions dropped into CaCl_2_ (0.1 M) to form beads; optimization by RSM identified 1% Na‐alg + 1.5% SM as optimal formulation	Encapsulation yield: 70.2% (Na‐alg) and 88.4% (Na‐alg + SM); postencapsulation viability 7.80 and 7.88 log CFU/mL, respectively	Viability remained stable after microencapsulation; long‐term storage stability not specifically evaluated	Higher survival in simulated gastric juice (pH 2.5, 120 min): 8.88 log CFU/mL (Na‐alg + SM) vs. 8.76 log CFU/mL (Na‐alg) and 7.72 log CFU/mL for free cells	Alginate–starch maleate composite improves protection under acidic gastric conditions, demonstrating a clear structure–function relationship between biopolymer composition and probiotic survival	Popović et al. (2021)
*Limosilactobacillus reuteri* ATCC 23272	Polysaccharide‐based bionanocomposite: inulin + pectin + sodium alginate matrix reinforced with MgO nanoparticles	Microencapsulation by extrusion–gelation into CaCl_2_ (0.1 M); polysaccharide prebiotic solution (1% w/v) containing MgO NPs (5 µg/mL), followed by microwave drying (400 W, 7.5 min)	Encapsulation efficiency 97.57%; viability after drying 99.37%; markedly higher survival compared to free cells	During 28 days storage, encapsulated cells showed reduced losses compared to free cells (2.56 log CFU/g at 4°C and 3.04 log CFU/g at 25°C prevented by encapsulation)	High survival under simulated gastrointestinal conditions (91.74%), whereas free cells lost 2.77 log CFU/g	Synergistic effect of prebiotic matrix and MgO nanoparticles enhances buffering capacity, limits acid and bile penetration, and improves probiotic survival and delivery to the colon	Mohamadzadeh et al. (2025)
*Limosilactobacillus reuteri* DPC16	Hempseed protein isolate (HPI) coencapsulated with Cyclocarya paliurus leaf extracts (protein–phenolic matrix)	Microencapsulation by protein–polyphenol complexation using HPI and C. paliurus leaf extracts at optimized 9:1 (w/w) ratio; drying to obtain stable powder	Encapsulation efficiency: 93.06%; post‐GITS viability 7.2 log CFU/g compared to 5.5 log CFU/g for free cells	Viability maintained above 6 log CFU/g after 120 days at −20°C, 4°C, and 25°C	Significantly higher survival after in vitro gastrointestinal tract simulation compared to free cells	Protein–phenolic interactions create compact matrices that reduce oxidation and acid penetration, enhancing probiotic stability and survivability during storage and GI transit	Lau et al. (2025)
*Limosilactobacillus reuteri* (strain not specified)	Pea protein microgel (MG)–reinforced low‐methoxyl pectin (LMP) hydrogel beads filled with pectic oligosaccharides (POS)	Two‐step encapsulation: preparation of pea protein microgels followed by entrapment into Ca^2^^+^‐crosslinked LMP hydrogel beads containing 0%–0.4% POS (MG/LMP/POS system)	Encapsulation efficiency increased with POS concentration; MG/LMP/POS0.4 showed the highest retention of viable cells after processing and simulated digestion	MG‐reinforced synbiotic hydrogel beads showed higher thermal and storage stability than MG alone	Higher survival throughout simulated oral, gastric and intestinal phases; most cells released under simulated colonic conditions after 48 h	Microgel‐reinforced hydrogel architecture and POS incorporation create a compact, dual‐network matrix that reduces cell leakage, improves resistance to upper GI stress, and enables colon‐targeted release	Yi et al. (2025)
*Limosilactobacillus reuteri* DSM 17938	Kudzu starch–hemp protein complex coacervates (KS–HP CC)	Complex coacervation (KS:HP ratio 1:2 w/w, pH 5.0) followed by spray drying (inlet 160°C, outlet 93°C)	High postprocessing viability; KS–HP CC retained ∼89% viability after simulated gastrointestinal digestion, ∼83.2% after 30 days storage at 25°C, and ∼94.2% after thermal treatment (50°C, 10 min)	Significantly improved storage stability over free cells and single‐material KS or HP microcapsules during 30 days at room temperature	Enhanced survival during simulated gastric and intestinal digestion compared to free cells and single‐wall systems	Protein–polysaccharide coacervate structure provides a compact protective matrix, improving resistance to gastrointestinal, thermal, and storage stresses while preserving probiotic functionality	Hamdi et al. (2025)
*Limosilactobacillus reuteri*	Low‐acyl gellan gum (LAG) + High‐acyl gellan gum (HAG) + Ca^2^^+^ (binary polysaccharide system)	Internal ionic gelation (emulsification with Span 80, Ca^2^^+^ crosslinking, Box–Behnken optimization)	Encapsulation efficiency up to 95.5%; viability after encapsulation up to 97.43% (optimized conditions)	Stable viable counts; maintained ≥8.02 log CFU/mL after simulated GI exposure	Viability decreased from 9.50 to 8.46 log CFU/mL after SGF (2 h) and to 8.02 log CFU/mL after SIF (2 h); free cells dropped to undetectable levels	Enhanced protection against gastric acid and bile salts; suitable for functional food applications	González‐Cuello (2025)
*Limosilactobacillus reuteri* DSM 17938	Alginate and chitosan–alginate matrices	Vibrating technology (extrusion‐based encapsulation); alginate beads with or without chitosan coating; CaCl_2_ gelation; optional freeze‐drying	High encapsulation efficiency (∼97%); maintained viability after encapsulation and freeze‐drying (≈100% survival immediately after lyophilization); reduced log losses under thermal, osmotic, and oxidative stress compared to free cells	Improved stability during 28 days of storage at 4°C (≈1 log reduction vs. ≈3 log for free cells); better protection in Ringer and NaCl solutions	Significantly enhanced survival under simulated gastrointestinal conditions (gastric pH 2.5 with pepsin and intestinal bile salts); chitosan–alginate capsules showed superior protection against bile stress	Preserved functional activity, including reuterin production and antimicrobial activity; maintained probiotic functionality while improving technological robustness	De Prisco et al. (2014)
*Limosilactobacillus reuteri* DSM 17938	Chia seed mucilage + sodium alginate	Electrohydrodynamic spraying (EHDA) and dripping mode; ionic crosslinking with Ca^2^^+^; nonthermal encapsulation	Viability ≈ 9.9 log CFU/mL after encapsulation and lyophilization; EE = 99.0 ± 0.01% (EHDA) and 99.0 ± 0.16% (DM)	High stability after freeze‐drying; maintained viability during powder formulation	Encapsulation matrix protected cells under simulated gastrointestinal conditions, improving survival compared to free cells	Successful incorporation into whey‐based functional powders; preserved probiotic viability and functional potential	Cid‐Córdoba et al. (2025)
*Limosilactobacillus reuteri*	Alginate beads coated with chitosan (alginate–chitosan microcapsules)	Extrusion–gelation technique: cell–alginate suspension extruded into CaCl_2_ solution to form beads, followed by chitosan coating via electrostatic interaction	Encapsulation efficiency > 90%; encapsulated cells showed significantly higher viability than free cells after encapsulation and thermal treatment	Improved stability during storage at 4°C; encapsulated cells showed lower viability loss compared to free cells over storage period	Encapsulated cells exhibited significantly higher survival under simulated gastrointestinal conditions (pH 2.0 with pepsin and bile salts) compared to free cells	Alginate–chitosan coating provided an effective physical barrier against acid and bile, enhancing probiotic survival and suitability for functional food applications	Chitprasert et al. (2012)
*Limosilactobacillus reuteri* DSM 17938	Chia seed mucilage + sodium alginate (binary polysaccharide hydrogel matrix)	Electrohydrodynamic spraying (EHDA); ionic crosslinking with CaCl_2_; nonthermal encapsulation followed by freeze‐drying	High postprocessing viability; encapsulation efficiency ≈ 98%–99%; viable counts remained > 9 log CFU/mL after encapsulation and lyophilization	Good storage stability of dried powders; encapsulated cells maintained viability during storage compared to free cells	Encapsulated cells showed significantly higher survival under simulated gastrointestinal conditions (gastric and intestinal phases) than free cells	Hydrogel microstructure provided effective protection against acidic and bile environments, enabling application in functional food systems	Cerón‐Córdoba et al. (2025)

Microbial counts are reported using the original units provided by each study (CFU/mL or CFU/g) depending on the food matrix and experimental design.

This integrative comparison in EE (Table 1) highlights the relationships between structure and function, showing how capsule architecture, polymer composition (wall material), and processing strategy can have a direct influence on *L. reuteri* viability, EE release behavior, and functional performance across different studies.

**FIGURE 1:**
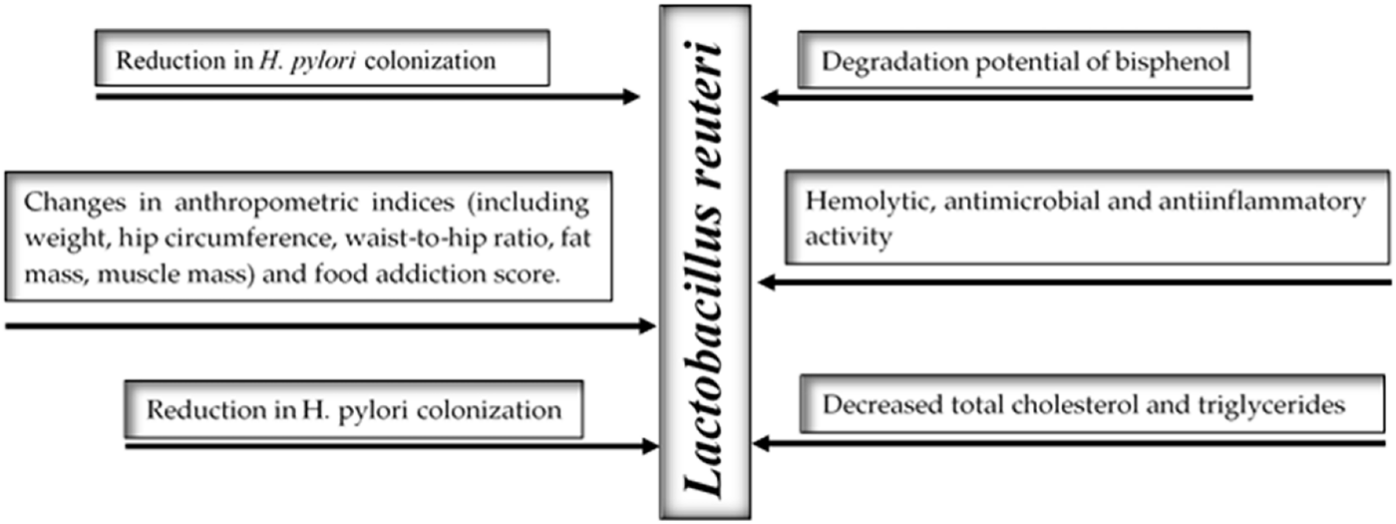
*Limosilactobacillus reuteri* strains in food matrices.

Current research on *L. reuteri* underscores the need to integrate strain physiology, wall material properties, technological outcomes, and functional performance. This review examines encapsulation studies involving *L. reuteri*, focusing on how strain‐dependent physiological characteristics affect EE, survival during processing and storage, and release under gastrointestinal conditions. It synthesizes evidence from various strains, materials, and encapsulation methods to identify new relationships across different food structures and functions (Figure 1).

**FIGURE 2:**
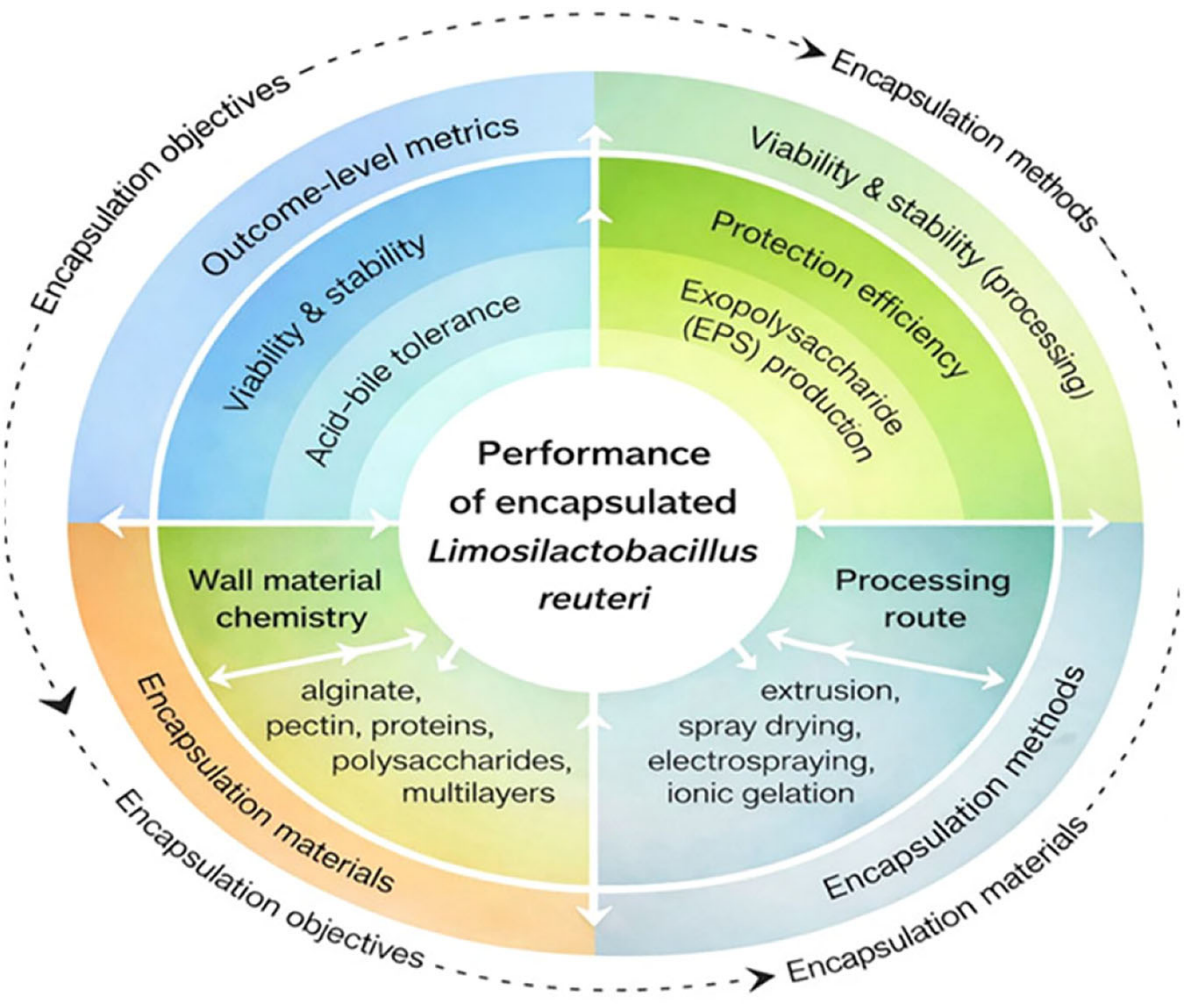
Summarizes this multilayer conceptual framework, illustrating how strain‐dependent physiological traits, encapsulation material properties, and processing strategies interact to determine the functional performance of encapsulated *Limosilactobacillus reuteri* in food matrices and gastrointestinal environments.

This framework links encapsulation design variables, such as the chemical composition of the coating material, encapsulation size, mechanical properties and process conditions, with strain‐dependent physiological characteristics, including EPS production and tolerance to gastric and intestinal acidity. This relationship clarifies outcome metrics, including the viability of encapsulated microorganisms and their stability during processing, storage and exposure to gastrointestinal stress. Figure 2 illustrates a structure–function approach for interpreting the selection of coating materials, encapsulation methods and processing conditions to maintain performance and functionality in food matrices.

## 2 Methodology of the Systematic Analysis

For this review, a systematic analysis of the scientific literature was conducted to examine both the functional activity and encapsulation of various *L. reuteri* strains. The primary objective was to identify the most extensively studied strains, prevalent encapsulation strategies, and the biological and technological mechanisms underlying their applications in food systems. This methodology facilitates the identification of knowledge gaps and technological challenges related to *L. reuteri* viability during processing, storage, and gastrointestinal transit.

### 2.1 Information Sources and Search Strategy

Relevant studies were identified through a structured search of three major scientific databases: Scopus, web of Science, and PubMed. The search utilized keywords included *L. reuteri*, encapsulation, probiotic viability, functional properties, anti‐inflammatory activity, spray drying, electrohydrodynamic spraying, and ionic gelation. Combinations of the search items were also employed. The database search was conducted in July 2025.

### 2.2 Selection and Synthesis

The initial database search identified approximately 180–220 records. Following title and abstract screening, nonrelevant publications and duplicate entries were excluded, resulting in 60–80 full‐text articles assessed for eligibility. Applications of predefined inclusion and exclusion criteria, which focused on strain‐specific data, food‐grade encapsulation materials, and quantitative performance metrics, yielded a final set of 30–40 studies for quantitative and integrative analysis (Figure 2).

The selected studies were critically analyzed and thematically organized according to three main criteria: (1) *L. reuteri* strain and associated functional characteristics, (2) encapsulation material architecture and processing technique, and (3) targeted technological or functional outcomes, such as gastrointestinal survival, storage stability, or antimicrobial activity. This thematic classification facilitated cross‐study comparisons and supported the identification of structure–function relationships across different encapsulation systems.

The selection process prioritized the most recent studies (2010–2025), also highlighting fundamental contributions and comparative data between strains and materials. This time frame was chosen because it encompasses the consolidation of probiotic encapsulation research, including the taxonomic reclassification of *L. reuteri* in 2020.

## 3 *Limosilactobacillus reuteri* (*LR*)

*L. reuteri* is one of the host‐associated bacteria found in different parts of the human body, including the gastrointestinal and urinary tracts, skin, and breast milk (Mu et al. 2018a). It has the ability to secrete bacteriocins, reuterin, and reutericyclin, which contribute to the control of gastrointestinal infections (Paternina et al. 2013). *L. reuteri* exhibits a wide range of health‐promoting effects, for example, hypocholesterolemic, antimutagenic, antimicrobial, and anti‐inflammatory activities. It also contributes to improved immune response and lactose digestibility, reduces colonization by *Helicobacter pylori*, and modulates anthropometric indices such as body weight, hip circumference, waist–hip ratio, fat mass, and muscle mass. Additionally, it shows potential for bisphenol degradation, displays moderate hemolytic activity, and helps lowering total cholesterol and triglyceride levels (Figure 2). Likewise, it has inhibitory effects on cytokines secretion, presenting beneficial effects on periodontal diseases (Alok et al. 2017). However, the effectiveness of probiotic microorganisms depends on the number of viable cells that might successfully colonize the intestine, and this viability depends of several factors like gastric acidity, digestive enzymes, and bile salts, all of them with negatively impact under probiotics stability (Juárez‐Tomás et al. 2015). Therefore, during the design of beneficial bacteria‐containing products, ensuring microbial stability throughout production processes and the product shelf life becomes a key technological requirement (Algaithi et al. 2022).

### 3.1 Functional Properties of *Limosilactobacillus reuteri* and the Role of Encapsulation Technologies

The health benefits of *L. reuteri* are well documented in the literature. However, the effectiveness of its antimicrobial, anti‐inflammatory, antioxidant, and antiobesity properties in food applications depends on bacterial survival during processing, storage, and gastrointestinal transit. The following sections outline the main functional activities of *L. reuteri* and examine its relationship with release systems and encapsulation.

#### 3.1.1 Functional Activity of *Limosilactobacillus reuteri* in Food Systems

*L. reuteri* has been incorporated into various food matrices due to its probiotic benefits, including balance of the gastrointestinal microbiota and metabolic regulation (Mu et al. 2018b). Studies have shown that encapsulating *L. reuteri* in fruit‐based beverages, such as mango pulp with maltodextrin and inulin, results in high survival rates (≈99%) and full cell viability during storage (Gámez et al. 2021). While antioxidant capacity was not directly evaluated, these findings highlight the importance of encapsulation strategies for maintaining probiotic viability in phenolic‐rich foods. Similarly, *L. reuteri* ATCC 55730 maintained high survival (≈98%) in a bean and oat‐based spread after 28 days at 4°C, supporting its use in plant‐based products (Barboza et al. 2012). Additionally, reuterin produced by *L. reuteri* has been shown to inhibit *Listeria monocytogenes* in cold‐smoked salmon (Montiel et al. 2014).

These studies reflect the potential of *L. reuteri* in food applications. However, its activity is sensitive to stress factors such as oxygen exposure, dehydration, and acidity (Baral et al. 2021). Encapsulation is therefore recommended to maintain the viability and functionality of *L. reuteri* during processing, storage, and gastrointestinal transit (Teymoori et al. 2024).

#### 3.1.2 Antimicrobial Activity

The antimicrobial activity of *LR* is primary attributed to the production of organic acids, bacteriocins, and strain‐specific metabolites, particularly reuterin (Banakar et al. 2023; Niamah et al. 2023). Reuterin exhibits broad‐spectrum antimicrobial activity against bacteria, yeasts, and fungi, making it especially relevant for food preservation (Jalali et al. 2024). The production and stability of reuterin are strongly influenced by environmental factors such as oxygen availability and pH. To address these limitations, several encapsulation strategies have been developed to protect both viable cells and antimicrobial metabolites. Juárez Tomás et al. (2015) demonstrated that encapsulating reuterin‐producing *L. reuteri* DSM 20016 in alginate‐based matrices effectively preserved antimicrobial activity by safeguarding the trapped cells and metabolites.

Jalali et al. (2024) further demonstrated that postbiotics derived from *L. reuteri* significantly inhibited the growth of *Escherichia coli* and *Staphylococcus aureus* on the surface of red meats. Collectively, these studies highlight the importance of encapsulation design in maintaining antimicrobial functionality and support the use of *L. reuteri* as a natural preservative in food systems. Nevertheless, additional research is still required to demonstrate and confirm its efficacy within food matrices and under processing conditions.

#### 3.1.3 Anti‐Inflammatory Activity

Different *LR* strains show anti‐inflammatory activity by modulating the immune response, such as downregulation of proinflammatory cytokines and stimulating anti‐inflammatory mediators (Werlinger et al. 2022). These effects are fundamental for maintaining the integrity of the intestinal barrier and immune homeostasis (Alam et al. 2022).

Most evidence for these anti‐inflammatory mechanisms comes from in vivo and in vitro studies using with nonencapsulated cells. While encouraging, practical application in functional foods depends on *L. reuteri*’s ability to survive gastrointestinal transit and reach the intestinal mucosa in sufficient quantities to exert immunomodulatory effects (Domingo and José 2017).

Therefore, encapsulation systems designed to protect cells against gastric acidity and bile salts are essential to preserve the anti‐inflammatory functionality of *L. reuteri* when delivered through food matrices.

#### 3.1.4 Antioxidant Activity

Oxidative stress arises from the accumulation of reactive oxygen and nitrogen species, and other free radicals, which damage cellular components through mechanisms such as DNA hydroxylation, lipid peroxidation, and protein denaturation. These processes contribute to the development of chronic degenerative diseases (Allameh et al. 2023; Reddy 2023). Antioxidants, including phenolic compounds, peptides, and probiotics have been extensively studied for their ability to mitigate oxidative damage and reduce disease incidence (J.‐Z. Wang et al. 2017).

Several *Lactobacillus* species, including *L. reuteri*, have demonstrated antioxidant effects through the production of bioactive metabolites and enzymatic defense mechanisms. Jalali et al. (2024) assessed the antioxidant activity of postbiotics derived from *L. reuteri* using the DPPH method and observed significantly higher radical scavenging activity postbiotics‐containing samples compared to controls. This effect was associated with elevated phenolic content, suggesting that metabolites produced by *LR* contribute to enhanced free radical neutralization. While comparative analyses indicated higher antioxidant activity for postbiotics from *L. rhamnosus*, the findings confirmed the antioxidant potential of *L. reuteri*‐derived postbiotics.

Encapsulation strategies have been explored to preserve probiotic viability and antioxidant functionality. Rodklongtan and Chitprasert (2017) investigated the coencapsulation of *L. reuteri* KUB‐AC5 with ascorbic acid during spray drying, using ascorbic acid as a complementary antioxidant to enhance cellular protection. Their findings showed high retention of ascorbic acid after spray drying and during refrigerated storage, with stable antioxidant activity evaluated by the ABTS method. However, storage at elevated temperatures decreased antioxidant capacity, underscoring the importance of proper storage. These findings demonstrate how encapsulation systems can help maintaining antioxidant functionality by protecting both probiotic cells and antioxidant compounds.

Additional studies have shown that fermentation with *L. reuteri* can increase antioxidant activity in food matrices by releasing phenolic compounds and bioactive peptides. Tyagi et al. (2021) reported that fermented brown rice had higher antioxidant activity attributed to the release of insoluble phenolic compounds during fermentation. Similarly, Begunova et al. (2021) showed that milk fermentation with *LR* produces antioxidant peptides through proteolytic activity. However, these studies mainly examined biological activity under controlled conditions and did not assess the stability of antioxidant functionality during food processing or storage.

Although the substantial evidence supporting the antioxidant potential of *L. reuteri* and its metabolites, few studies have examined how encapsulation contribute to preserving this functionality under food processing and storage.

#### 3.1.5 Antiobesity Potential and Technological Implications

Obesity is a multifactorial metabolic disorder that is strongly associated with gut microbiota dysbiosis, chronic inflammation, and disruptions in glucose and lipid metabolism (Ahmed and Konje 2023). Several studies have shown that different strains of *L. reuteri* produce antiobesity effects in animal models, such as reductions in body weight gain, adiposity, insulin resistance, and inflammatory markers under high‐fat or high‐fructose dietary conditions (Hsieh et al. 2013; Larsen et al. 2023; Tang et al. 2025).

These beneficial effects have been associated with the modulation of adipogenic and lipogenic gene expression, enhance glucose tolerance, reduction of systemic inflammation, and protection against nonalcoholic fatty liver disease (Di Porzio et al. 2023; Kim et al. 2022; Wu et al. 2025). Notably, most of these studies have been conducted using nonencapsulated cells administered in controlled experimental conditions.

From a technological perspective, the translation of the reported antiobesity effects of *L. reuteri* into functional food applications remains limited by challenges related to probiotic survival, stability, and targeted intestinal delivery. Encapsulation strategies are therefore critical for maintaining cell viability and ensuring consistent delivery of *L. reuteri* to the intestine, which is essential to achieve its metabolic benefits in real food systems.

To facilitate comparison between studies, Table 2 compiles the literature analyzed in the section Functional Properties of *L. reuteri* and the Role of Encapsulation Technologies, indicating whether the functional activity was experimentally evaluated and the presence of encapsulation strategies in food matrices.

**TABLE 2:** Summary of studies evaluating functional activity and encapsulation strategies of *Limosilactobacillus reuteri* in food‐related systems.

*L. reuteri* strain	Food matrix/system	Encapsulation strategy	Functional activity or experimentally evaluated	Outcome on functional activity	References
*L. reuteri* ATCC 55730	Legume‐based spread	Not applied	Yes	High probiotic survival during refrigerated storage, supporting functional performance in plant‐based matrices	Barboza Martínez et al. (2012)
*L. reuteri* (not specified)	Cold‐smoked salmon	Not applied	Yes (antimicrobial)	Reuterin production effectively inhibited Listeria monocytogenes	Montiel et al. (2014)
*L. reuteri* DSM 20016	Model system	Alginate‐based encapsulation	Yes (antimicrobial)	Encapsulation preserved antimicrobial activity by protecting viable cells and entrapped metabolites	Juárez‐Tomás et al. (2015)
*L. reuteri* KUB‐AC5	Powdered food model	Spray drying with lactose and ascorbic acid	Yes (antioxidant)	Coencapsulation preserved antioxidant activity and improved stability during storage	Rodklongtan and Chitprasert (2017)
*L. reuteri* ATCC 53608	Mango pulp‐based beverage	Spray drying with maltodextrin and inulin	No	Encapsulation improved probiotic stability, supporting preservation of functional potential	Gámez et al. (2021)
*L. reuteri* (not specified)	Meat‐based system	Not applied	Yes (antioxidant)	Postbiotic application enhanced antioxidant capacity of the meat matrix	Jalali et al. (2024)

### 3.2 Strain‐Specific Differences

Several probiotic strain varieties have been studied in the pharmaceutical and food industries due to their high functional and therapeutic potential, demonstrating substantial genomic diversity and adaptability to different ecological niches Some strain‐dependent characteristics are summarized in Table 3, which compiles the most frequently studied *L. reuteri* isolates and their functional properties. Among the most extensively studied strain, particularly in pediatric populations, the strains DSM‐17938 have received considerable attention. Several reports demonstrated its effective gastrointestinal tract colonization producing a reduction in diarrhea events related to rotavirus infection (Sun et al. 2023).

**TABLE 3:** *Limosilactobacillus reuteri* strains with functional capabilities.

*Lactobacillus Reuteri* strain	Functional activity	References
FCQHC8L6 y FYNDL13	Anti‐inflammatory and Antimicrobial	Lin et al. (2023)
BR120	Probiotic, anti‐inflammatories, radical scavengers, acid tolerance, bile tolerance and intestinal adhesion	Ju et al. (2023)
ATCC 53608	Antimicrobial activity against viruses, fungi, protozoa and gram‐positive and gram‐negative bacteria	Ma et al. (2023)
ATCC PTA5289	Inhibits the acid tolerance response in oral bacteria	Boisen et al. (2023)
DSM 17938	Colonize the gastrointestinal tract effectively and shorten the duration of watery diarrhea associated with rotavirus infection time significantly	Sun et al. (2023)
NCIMB 30242	Reduced total cholesterol (TC)	Liu et al. (2023)
DSM 20016 y DSM 17938	Antimicrobial characteristics, such as antifungal activity and a broad‐spectrum of activity against Gram‐positive and negative bacteria	Rodrigues et al. (2023)
DSM17648	Reduction in H. pylori colonization	
DSM 17938	Antimicrobial activity	Zhang et al. (2022)
ATCC 53608	Antimicrobial activity	Soltani Lak et al. (2021)
DSM 17648	Reduction in H. pylori colonization	Yang et al. (2021)
DSM 17648	Reduction in H. pylori colonization	Dargenio et al. (2021b)
ATCC 23272	Probiotic	Karimi et al. (2021)
KUB‐AC5	Probiotic	Diệp Huy Vũ et al. (2021a)
B2	Antimicrobial activity and Acid resistance and Bile Salt Tolerance	Popović et al. (2021)
B‐14171.	Inhibit the growth of pathogenic bacteria and improve the immune system of the host.	Mis Solval et al. (2019)
E81	Effect against H. pylori infection	Ceylan et al. (2019)
ATCC 55730	Degradation potential of bisphenol	Ju et al. (2019)
B‐14171	Antimicrobial	Mu et al. (2018)
ATCC 55730 y ATCC PTA 5282	Antimicrobial and anti‐inflammatory properties	Kaur et al. (2018)
ATCC 55730	Treatment of diarrhea, hernia and intestinal backflow	Indrio et al. (2011)
DSM 20016T; ATCC 23272	Biotherapeutic potential	Juárez Tomás et al. (2015)
DSM17648	Reduction in H. pylori colonization	Holz et al. (2015)
ATCC PTA 6475 y ATCC PTA 5289	Antimicrobial activity	Jones and Versalovic (2009)
NCIMB 30242	Reduced total cholesterol (TC)	Casas and Dobrogosz (2000)
CRL 1098	Decreased total cholesterol and triglycerides	Taranto et al. (1998)

Clinical studies in orthodontic patients showed that tablets combining *L. reuteri* DSM17938 and PTA5289 reduced the growth of *Streptococcus mutans*, improving indicators such as plaque, gingivitis, and gingival bleeding, although without reaching statistical significance. In another approach, this strain (DSM17938 and PTA5289) has shown to interfere with the adhesion of *S. mutans* to hydroxyapatite‐coated surfaces demonstrating anticaryotic potential, whereas ATCC 55730 displayed antimicrobial and anti‐inflammatory properties (Kaur et al. 2018). Additional strains, such as ATCC 53608, are widely used as they are considered broad‐spectrum against various pathogenic microorganisms including viruses, fungi, protozoa, and bacteria (Ma et al. 2023; Soltani Lak et al. 2021). On the other hand, CRL 1098 has exhibited a decrease in triglycerides and cholesterol levels, thus improving metabolic health. Similar results were obtained with the NCIMB 30242 strain, which has shown total cholesterol levels reduction in clinical studies (J. Liu et al. 2023).

KUB‐AC5 stands out for its application in food matrices as result of its excellent gastrointestinal colonization ability and because its viability is preserved after its spray drying which courage its probiotic applications at industrial level (Diệp Huy Vũ et al. 2021). Strains such as BR120, FCQHRCL6, and FYNDL13 have been identified in internal datasets or preliminary investigations for possessing multifunctional properties including probiotic activity, anti‐inflammatory effects, radical scavenging capacity, tolerance to gastric acidity, and adhesion to intestinal epithelial cells. Among the most relevant strains with health benefits can be find the DSM 17648, which can reduce *Helicobacter pylori* colonization improving digestive wellness (Dargenio et al. 2021; Yang et al. 2021). Other strains, such as B‐14171 and E81, also exhibit antimicrobial capacity and contribute to the modulation of the immune response, highlighting the breadth of mechanisms by which *L. reuteri* strains exert their functional roles (Ceylan et al. 2019; Mis Solval et al. 2019).

CRL1324 is a microbial strain designed to enhance vaginal health. It helps maintaining viability in vaginal fluid and inhibiting the proliferation of *Streptococcus agalactiae*, which can induce the release of cytokines causing inflammation in vaginal tissue and the subsequent decreasing of protective microbiota (Juárez Tomás et al. 2015). In the same study, it was demonstrated that the probiotic potential of *L. reuteri* differs depending on the strain used.

In brief, the probiotic potential of *L. reuteri* is not uniform and rather is highly strain‐dependent, with each isolate exhibiting specific applications. Although strain diversity is desirable because this expands the applicability of these bacteria, at the same time it faces a challenge. Mainly because every microbial strain presents different stability and viability under certain conditions making their study or applications into foods or supplements difficult. To overcome these challenges, encapsulation has emerged as strategy to improve preservation of the strain functional activities and to control its release into the diana host.

### 3.3 Encapsulation of *Limosilactobacillus reuteri*

Different factors can affect the stability of probiotics in food products including food properties, processing parameters, and microbial characteristics (Algaithi et al. 2022). For probiotic microorganisms to reach the intestinal mucosa, stomach acidity is an almost insurmountable barrier for microorganisms ingested in drinks and foods, thus protecting the intestine against infections. Consequently, the number of viable microorganisms that survive this barrier is often too low to exert a significant beneficial effect.

To address these stability challenges and provide additional protection against environmental stress in the gastrointestinal tract, microencapsulation techniques are considered among the most effective strategies for enhancing probiotic survival during processing and storage, thereby ensuring their activity in the intestine (J. Wang et al. 2017). To overcome this obstacle, encapsulation can be defined as a process in which liquid, solid, or gaseous particles referred to core, active substance, internal phase, or payload are enclosed within a porous polymeric film (Araneda and Valenzuela 2009), forming a capsule, wall material, membrane, carrier, or shell (Gibbs et al. 1999). It can also be regarded as a unique method of packaging, isolating, and storing materials at the micro or nanoscale for subsequent controlled release or to prevent unwanted reactions between components (Astray et al. 2009).

**FIGURE 3:**
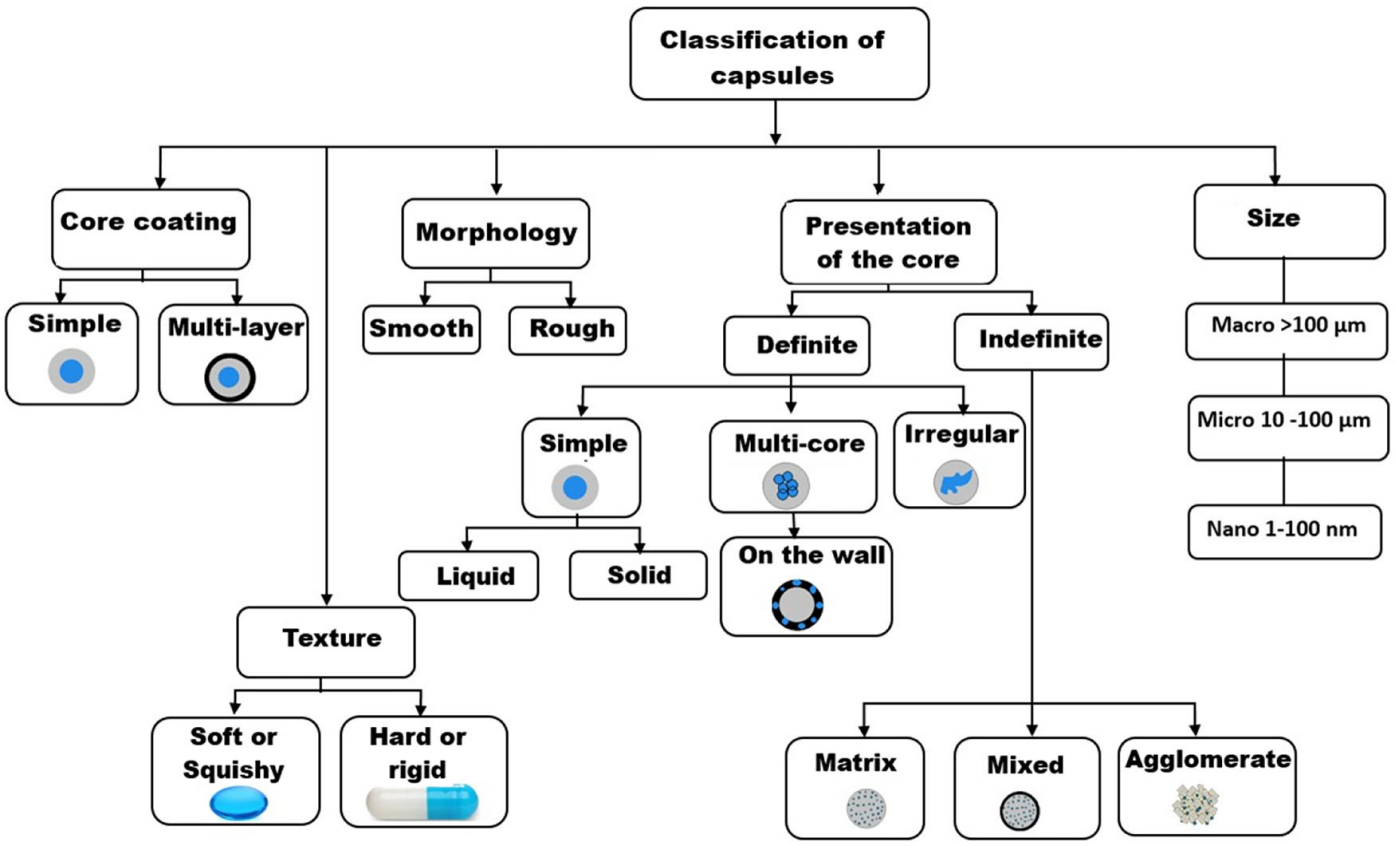
Different classifications of capsules.

As shown in Figure 3, capsules can also be classified according to structural criteria, such as the core coating (monolayer or multilayer), texture (soft or rigid), morphology (smooth or rough), and core presentation (defined or undefined, with matrix, mixed, or agglomerated). Size is also a determining factor, ranging from macrocapsules (> 100 µm) to microcapsules (10–100 µm) and nanocapsules (1–100 nm). Among these, matrix‐type capsules are the most widely used in food and pharmaceutical systems, as they allow for smaller diameters and a homogeneous distribution of the active compound (Sosnik 2014; J. Wang et al. 2017). This framework supports the interpretation of the diverse encapsulation methods used for *L. reuteri*, which often result in matrix‐based systems when employing alginate or its blends.

This technique allows individual coating of materials to protect them from harmful external factors, creating a microenvironment capable of regulating interactions between the core and its surroundings (Borgogna et al. 2010). The capsule wall can provide protection against heat, light, moisture, oxygen, and pH variations, as well as improve processing resistance, packaging stability, flavor, aroma, nutritional value, and appearance of the final product (Yañez Fernández et al. 2002). Current research continues focusing on developing various macro‐, micro‐, and nanoencapsulation techniques that allow the delivery of probiotics in adequate quantities to confer health benefits, giving place to multifunctional foods (Liu et al. 2016).

The different encapsulation techniques for *L. reuteri* are divided into physicochemical and physical methods, each with its own advantages and limitations. Chemical methods include interfacial polymerization or molecular inclusion, although the use of nonbiodegradable materials limits their application in foods. Physicochemical techniques, including coacervation and liposome entrapment, provide tailored release and protection of sensitive compounds, although their stability and cost remain challenging.

Physical methods are the most widely applied in probiotics because of their scalability and food‐grade status. Spray drying is the most widely used technique due to its low cost and industrial viability. However, its limitation is caused by thermal stress, leading to a reduction in cell viability unless protective carriers are added. Lyophilization, on the other hand, guarantees high survival rates and long‐term stability, but requires more time and resources. While extrusion with ionotropic gelation, when using alginate or gums, effectively protects *L. reuteri* from gastrointestinal stress, the capsule's size and mechanical weakness can limit its consumer acceptance. More recently, electrohydrodynamic spraying (electrospraying) has gained attention for producing micro‐ and nanocapsules with controlled morphology and high EE, although scaling the process remains difficult.

Taken together, the method selected depends on striking a balance between probiotic survival, release behavior, compatibility of coating materials, and feasibility for industrial application.

In addition to quantitative outcomes summarized in Table 1, it is relevant to examine the mechanistic basis by which encapsulation materials influence stress tolerance and functional performance of *L. reuteri*.

In addition to enhancing survival rates, encapsulation materials affect the functional performance of *L. reuteri* through multiple interconnected mechanisms. Dense or multilayer polymeric matrices, such as alginate‐ chitosan systems or protein polysaccharide coacervated, reduce membranes damage during gastrointestinal transit (Anal and Singh 2007; Cook et al. 2012; de Prisco and Mauriello 2016). Furthermore, polymers with buffering capacity or antioxidant properties can stabilize the local microenvironment, thereby supporting membrane integrity and enzymatic activity. Although encapsulation does not directly enhance adhesion to intestinal epithelium, it preserves related functions after release. Sustained metabolic activity, including the production of antimicrobial metabolites such as reuterin and other postbiotic compounds, may be directly supported by encapsulation strategies that limit oxidative stress and dehydration‐induce cellular damage (Juárez‐Tomás et al. 2015; Rodrigues et al. 2023).

Taken together, the encapsulation methods for *L. reuteri* vary widely in terms of cost, scalability, and protective capacity. Spray drying and ionic gelation remain the most widely adopted, while emerging methods such as electrospraying provide new opportunities but are still far from industrial application.

Regardless of laboratory‐scale results, global consumption of probiotic‐containing foods has increased substantially in recent years. Market analysts project that the global probiotics sector will continue to expand toward 2030, reflecting growing demand for functional foods and dietary supplements (Grand View Research 2024). This sustained growth underscores the need for formulation strategies that enable industrial‐scale implementation. In this context, advances in encapsulation technologies are critical to ensuring stability, functional performance, and regulatory compliance of commercially viable food products containing *L. reuteri* strains.

#### 3.3.1 Industrial and Scaling Considerations for Encapsulated *Limosilactobacillus reuteri*

Although numerous encapsulation strategies have improved viability and function of *LR* under controlled laboratory settings, their successful application in real‐world food systems depends on maintaining viability and stability under industrial processing conditions. Despite promising results reported at the laboratory scale, the industrial implementation of encapsulated *L. reuteri* remain challenging, with coating materials as one of the most critical factors. Encapsulation matrices must be food grade, GRAS‐certified, cost‐effective, and compatible with the physicochemical and sensory properties of the food matrix in which they will be incorporated. Polymers such as alginate, certain starch derivatives, proteins, gums, waxes, and some prebiotics meet these requirements and are suitable for large‐scale applications.

On the other hand, maintaining the stability of both the coating material and the microorganism during processing is a significant challenge. Food processing methods, such as spray drying, mixing, pumping, extrusion, and heat treatments, expose probiotic microorganisms to stresses like shear, dehydration, temperature changes, and oxygen, which can compromise their viability. While encapsulation techniques like electrospraying and nanoencapsulation offer strong protection at small scales, their scalability, performance, and cost are limiting. Spray drying and ionic gelation remain to dominate industrial applications due to their robustness and economic effectiveness, but they require careful optimization to preserve microorganism viability and stability.

Based on the above, future industrial implementation should prioritize encapsulation systems that balance EE, stability, and feasibility with production process scalability, regulatory acceptance, and stability in real‐world food matrices. Bridging the gap between laboratory results and industrial viability is essential for the successful commercialization of encapsulated *L. reuteri* in functional foods.

**FIGURE 4:**
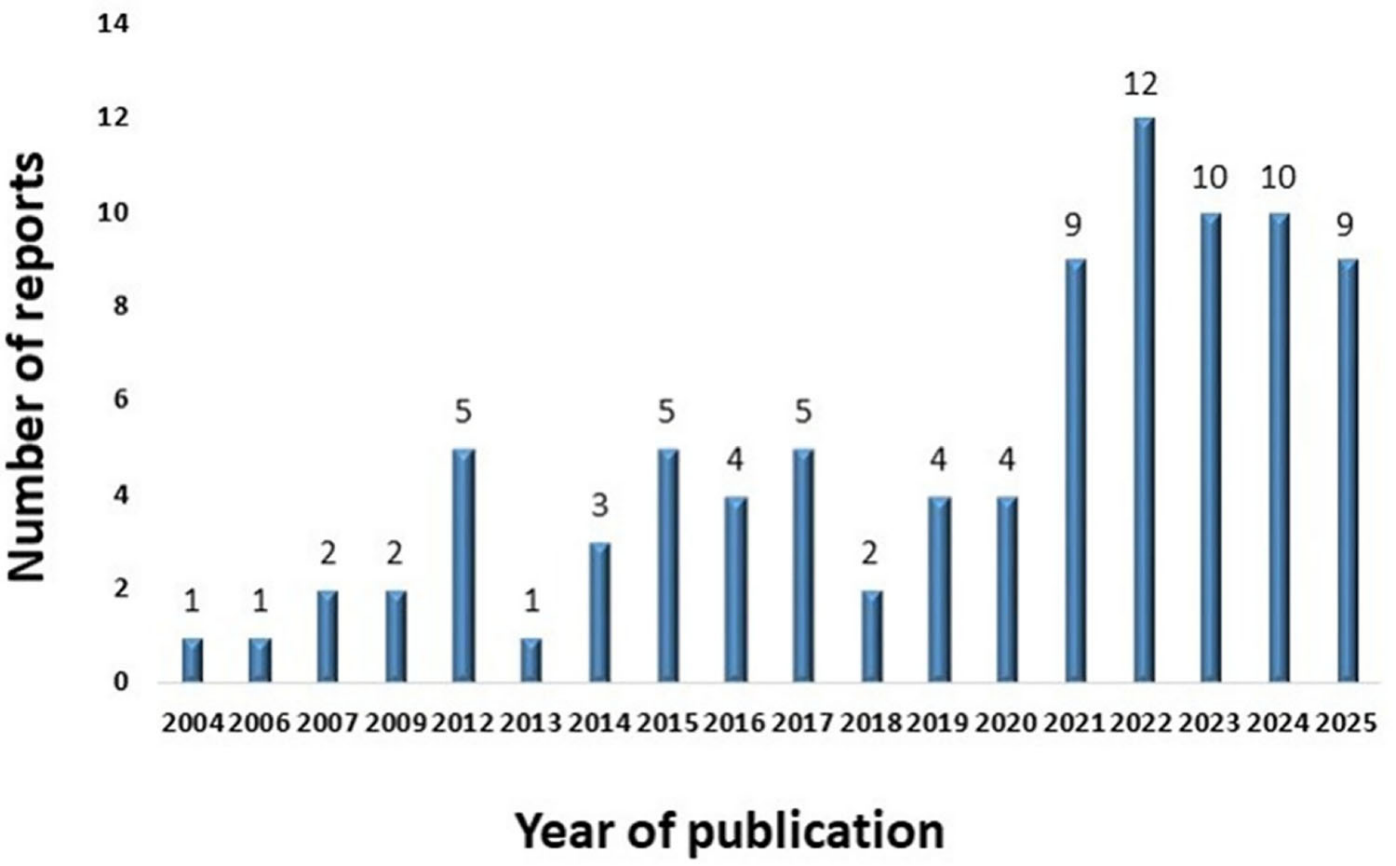
Articles related to the encapsulation of *Lactobacillus reuteri* (Scopus database; keywords: “encapsulation” and “*Lactobacillus reuteri*”; year‐individual: 2004–2025) and limiting it to articles only.

In recent years, studies on the encapsulation of *L. reuteri* have shown a steady upward trend (Figure 4). Since the first report in 2004, the field has progressed slowly, with only one or two publications per year until 2015. Subsequently, a gradual increase was observed and by 2020, the number of contributions had increased dramatically, reaching an average of 10 per year for the past 5 years, according to the Scopus database. This increment reflects the growing scientific interest in *L. reuteri* as a probiotic model, as well as the diversification of encapsulation methods, which include electrospray, ionic gelation, and other techniques using different biopolymers. Notably, the sharp increase after 2020 could also reflect advances in biopolymer science and the urgent demand for stable probiotics. The increase in work related to probiotics encapsulation is the results of demonstration that the encapsulation traps bacteria in a coating matrix, preventing their exposure to harmful environmental conditions and improving their viability. The steady growth of publications also shows that *L. reuteri* is no longer studied in isolation but rather as a reference strain to test emerging encapsulation technologies. This perspective helps explain its recurrent presence in formulations aimed at improving probiotic stability and functionality.

As shown in Table 4, the most commonly used coating materials for *L. reuteri* encapsulation include biopolymer blends such as sodium alginate, calcium alginate, maltodextrin, and others, mainly applied through ionic gelation techniques. Sodium and calcium alginate predominate due to their low cost, biocompatibility, and GRAS status. However, their combination with proteins (e.g., whey protein or hemp seed protein) or polysaccharides (e.g., inulin or plant mucilage) generally results in higher EE. Accordingly, the physicochemical compatibility between alginate and other complementary biopolymers improves the survival of *L. reuteri* under gastrointestinal stress, as well as its textural and sensory properties when incorporated into food matrices.

**TABLE 4:** Coating materials used in the encapsulation of *Lactobacillus reuteri*.

Core materials	Encapsulation efficiencies (EE)	References
Protein isolates	93.06	Lau et al. (2025)
Beads of alginate hydrogel embedded with gellan fluid gel	86.64	Najafpour et al. (2025)
Whey proteins and gum Arabic	82.11	Atta et al. (2025)
Mucin and hempseed protein isolate	Not reported	Lau and Quek (2025)
Hydrogel formed by hyaluronic acid modified with thiol groups of L‐cysteine	38.15	Yang et al. (2025)
Polysaccharide‐based bionanocomposite. Inulin, polydextrose, and pectin were utilized as prebiotics, and magnesium oxide nanoparticles as reinforcing agent in the microgel structure	99.37	Mohamadzadeh et al. (2025)
Inulin and maltodextrin	43.27	Ceron‐Cordoba et al. (2025)
Whey protein isolate or sodium caseinate (Cas), followed by pectin	92	Rodríguez et al. (2025)
Chitosan and Ca‐alginate beads	92.5	Jeznienė et al. (2024)
Whey protein concentrate and gum arabic	67	Teymoori et al. (2024)
Sodium alginate and tomato seed mucilage	91.54	Ganje et al. (2024)
Alginate or pectin followed by polymeric coating (with whey protein concentrate or chitosan)	91	Mosquera‐Vivas et al. (2024)
Alginate‐inulin	Not reported	Yadav et al. (2024)
Rice protein, pea protein and inulin	96.99	Mudgil et al. (2024)
Gelatin and pregelatinized starch, maltodextrin and Cheese whey permeate	50.53	Zimmermann et al. (2024)
Binary matrix of inulin and maltodextrin	91.35	Cerón Córdoba et al. (2024)
Cruciferin/alginate	Not reported	Akbari et al. (2023)
Chitosan‐coated alginate‐inulin matrix	95.85	Parsana et al. (2023)
Skim milk and infant formula	90	Alves Gragnani Vido et al. (2023)
Binary matrix composed of inulin and maltodextrin	89.76	Jurado‐Gámez et al. (2023)
Skim milk, sodium glutamate, polyvinylpyrrolidone, maltodextrin and gelatin	Not reported	Wang et al. (2023)
Sodium alginate and sesame seed oil	89.22	Javed et al. (2023)
Alginate‐konjac gum	Not reported	Rodrigues et al. (2023)
Acacia gum	Not reported	do Nascimento et al. (2023)
Alginate	97	Abuqwider et al. (2023)
Pequi oil	96	Cedran et al. (2022)
fish skin gelatin		Zhang et al. (2022)
Sodium hyaluronate	79	Li et al. (2022)
Isolated lignin complex from whey protein (WPI) and lignin	100	Diệp Huy Vũ et al. (2021b)
Hydrophobic surfactant into the MCT	84	Marefati et al. (2021)
pomegranate (*Punica granatum*)	46	Bron et al. (2021)
Sunflower oil, Skim milk powder	98.93	Kil et al. (2020)
novel enzymatically modified resistant starch type III	86.73	Khan et al. (2020)
yellow passion fruit (*Passiflora edulis* Sims f. *flavicarpa* Deg.) and gelatin	99	Santos Monteiro et al. (2020)
PVA	78	Ceylan et al. (2019)
Gum arabic	63	Guergoletto et al. (2017)
gelatin	24.4	Guergoletto et al. (2017)
Holy basil essential oil	47.73	Rodklongtan and Chitprasert (2017)
Milk powder	74.91	Liu et al. (2016)
xanthan–gellan gum	72,5	Juárez Tomás et al. (2015)
Aluminum carboxymethyl cellulose–rice bran	99.71	Chitprasert et al. (2012)
Palm oil was of food grade	99	Chitprasert et al. (2012)
Mixtures of sodium alginate with other biopolymers	83.08	Akbari et al. (2023), Javed et al. (2023), Parsana et al. (2023), Rodrigues et al. (2023), Algaithi et al. (2022), Rodrigues et al. (2022), Popović et al. (2021), Karimi et al. (2021), Huang et al. (2021), Nakkarach and Withayagiat (2018), Atia et al. (2016), De Prisco et al. (2015), Rodklongtan and Chitprasert (2017), Petraitytė and Šipailienė (2019)
Sodium alginate	70	Ebrahimi Monfared et al. (2022), Qaziyani et al. (2019), Muthukumarasamy et al. (2006)
Maltodextrin	57	Guergoletto et al. (2017), Schell and Beermann (2014)
Blends of maltodextrin with other biopolymers	89.75	Gámez et al. (2021), Breitenbach et al. (2016)
Whey protein isolate, sucrose	68.4	Sompach et al. (2022)
Whey	0.54	Jantzen et al. (2013)

These findings emphasize that the choice of wall material is as crucial as the encapsulation technique by itself. Consistent with the trends summarized in Table 4, several studies have shown that combining two or more coating materials further enhances encapsulation performance and probiotic viability. For example, Lau et al. (2025) developed a novel microencapsulation system using hemp seed protein isolate (HPI) and *Cyclocarya paliurus* leaf extracts (CP) for the targeted delivery of *L. reuteri* DPC16 to the human intestine. The optimized HPI/CP (9:1, w/w) formulation achieved the highest EE and maintained 93.06% survival in an in vitro gastrointestinal simulation (GITS) during 120 days of storage at −20°C, 4°C, and 25°C. After in vitro GITS, encapsulated *L. reuteri* DPC16 cells maintained mean cell counts of 7.2 log CFU/g, higher than those observed in unbound cells (5.5 log CFU/g; *p* < 0.05). In line with this evidence, several studies have shown that combining two or more coating materials further enhances encapsulation performance and probiotic viability. Confocal microscopy confirmed its robust survivability in the HPI/CP (9:1) matrices, with viability remaining above 10^6^ CFU/g after 120 days. These findings highlight the potential of HPI and CP formulations for developing functional foods and nutraceuticals with enhanced probiotic stability and survivability.

Besides, the alginate remains one of the most widely used coating materials due to its biocompatibility, low toxicity, cost‐effectiveness, and minimal impact on sensory attributes (Tekin et al. 2023). A key feature of alginate is its ability to form stable gels in the presence of divalent cations such as Ca^2^^+^, which is the fundamental mechanism exploited in ionotropic gelation techniques. Despite these advantages, when Alginate is used alone, encapsulation efficacies may be moderate, or can be relatively low (70%) and this makes it necessary to combine it with other biopolymers such as whey protein, chitosan, inulin, or plant mucilages (see Table 4) to increase efficiencies until averaged values of around 83% (Pereira Silveira et al. 2023).

On the other hand, regarding the encapsulation methods, they are selected based on the desired capsule size and type, the physical and chemical properties of the materials, and the expected release mechanism (Culas et al. 2024). Encapsulation methods can be classified as physical (e.g., spray drying, lyophilization, fluidized‐bed coating, extrusion, coextrusion and melt extrusion), physicochemical (e.g., molecular inclusion and liposomal encapsulation), or chemical (e.g., coacervation, ionic gelation and interfacial polymerization).

**FIGURE 5:**
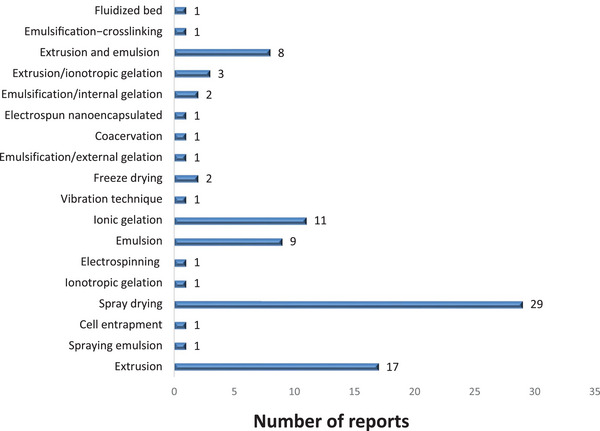
Trend in the number of publications related to *Lactobacillus reuteri* encapsulation methods (Scopus database; keywords: “encapsulation” and “*Lactobacillus reuteri*,” year‐individual: 2004–2025) and limiting it to articles only.

It is important to mention that every encapsulation technique has specific advantages and limitations. For example, spray drying remains the most scalable method; however, its high‐temperature exposure can reduce viability in sensitive strains. In contrast, EHDA including electrospray, avoids intense temperature variations and allows high particle size and morphology control, enhancing microbial integrity preservation; however, it presents limitations in terms of industrial scalability and cost‐effectiveness. Despite all the above, it has been observed that spray drying is emerging as one of the most widely used techniques for encapsulating microorganisms, followed by extrusion and ionic gelation (Figure 5). Particularly, ionic gelation has been extensively used due to its biocompatibility and the production of capsules with a broad size distribution; even so, it exhibits lower mechanical resilience, which could affect viability during storage or when incorporated into liquid food matrices.

Recently, coencapsulation strategies have also been explored to improve probiotic survival. These approaches usually combine probiotics with proteins, polysaccharides, or prebiotics through established methods like spray drying or ionic gelation. Promising results have been reported, for example with rice protein or pea–inulin mixtures, which achieved survival rates above 90% for *L. reuteri* DSM 17938 after spray drying. In addition to protecting the cells during GITS, these hybrid matrices seem to provide extra resistance to thermal and osmotic stress compared to single‐material systems.

**FIGURE 6:**
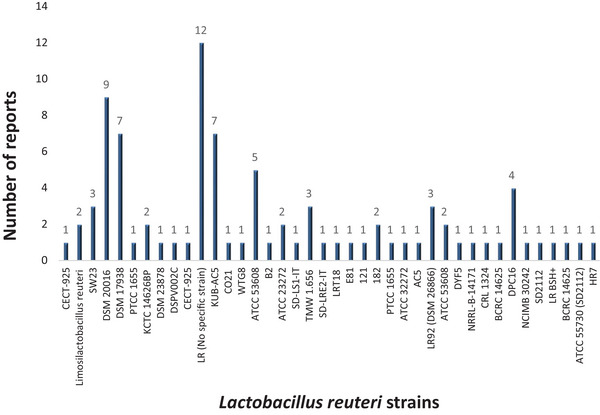
Articles related to encapsulated strains of *Lactobacillus reuteri* (Scopus database; keywords: “encapsulation” and “*Lactobacillus reuteri*,” year‐individual: 2004–2025) and limiting it to articles only.

Regarding *Lactobacillus reuteri* strains reported in encapsulation procedures, the analysis of the articles consulted for this review, demonstrated that the most studied strain is DSM 20016 due to its antimicrobial characteristics, like antifungal activity and effect against Gram positive and negative bacteria (Juárez‐Tomás et al. 2015; Rodrigues et al. 2023) and which has been used with different encapsulation matrices (Figure 6). In addition to the above, in a related study, *L. reuteri DSM 20016* produced reuterin in situ (46.67 mmol/L) in alginate–konjac gum film‐forming solution through anaerobic glycerol bioconversion. After film production by extrusion, both *L. reuteri* cells and reuterin were successfully entrapped (98% and 67.6%, respectively). However, the metabolites responsible for these inhibitory effects need to be studied (Juárez‐Tomás et al. 2015). Moreover, it has been shown that the DPC16 strain colonizes human intestine due to the increased expression of proteins in the gastrointestinal mucosa, which supports its application in therapeutic supplements (Lau et al. 2025). Another very important studied strain is KUB‐AC5 for its probiotic viability (Diệp Huy Vũ et al. 2021a).

In addition to these strains, encapsulation of *L. reuteri* CRL 1324 by ionic gelation extrusion with xanthan and gellan as coating materials, followed by lyophilization with lactose or skimmed milk as cryoprotectants, has also been investigated. This approach improved bacterial survival during prolonged storage at 4°C and increased viability in simulated vaginal fluid. The encapsulated cells also retained their functional properties by inhibiting the growth of *Streptococcus agalactiae*. Moreover, reuterin‐producing strains (DSM 20016 and DSM 17938) incorporated into alginate–konjac gum edible films were reported to inhibit foodborne pathogens (*B. cereus*, *C. perfringens*, *P. aeruginosa*) and spoilage fungi (*F. oxysporum*, *C. gloeosporioides*, *A. alternata*, *P. digitatum*), indicating broader antimicrobial potential. Overall, this review highlights the strains with the most significant number of studies in various polymer matrices and encapsulation methods, aiming to better understand current trends in encapsulation and the applications of this probiotic microorganism.

### 3.4 Regulatory

Beyond the technical progress in encapsulation, the relevance of *L. reuteri* depends largely on whether these advances can be translated into products that are safe, compliant with regulation, and attractive for the market.

#### 3.4.1 Regulatory Overview

Regarding the regulations governing the use and application of probiotic microorganisms, the European Food Safety Authority (EFSA) applies the concept of Qualified Presumption of Safety (QPS) as a tool to simplify the safety assessment of microorganisms. If a species appears on the QPS list, as is the case with *L. reuteri*, it is considered safe unless there is evidence to the contrary (EFSA 2023). However, to date, there are regulatory barriers to health claims. In this regard, both the Health Claims Regulation (EC 1924/2006) and the Novel Foods Regulation (EU 2015/2283) require solid clinical evidence of their functionality, and the encapsulation of *L. reuteri* still requires additional studies to demonstrate stability, viability, bioavailability, safety, and functional benefits (EFSA 2024).

In addition to the above, the Food and Drug Administration in the United States, classifies probiotic microorganisms as dietary supplements or functional foods, depending on their formulation and intended use. Concerning their labeling, it is prohibited to suggest therapeutic effects unless supported by scientific evidence. This has prompted researchers and industry to adopt more rigorous evaluation methods. Furthermore, the US Pharmacopeia has recently highlighted that conventional toxicological approaches are not always suitable for evaluating live microorganisms (Merenstein et al. 2023; Roe et al. 2022).

#### 3.4.2 Security, Regulations and Emerging Technological Trends (*Limosilactobacillus reuteri* Encapsulated)

From a safety perspective, the regulatory assessment of probiotic microorganisms, including *L. reuteri*, is primarily strain‐specific due to differences in genomic backgrounds and functional characteristics, which impact antibiotic resistance profiles. In this regard, the European Union's QPS provides a basis for microbial preassessment and several EFSA opinions detail the evaluation of various *L. reuteri* strains. However, labeling specific functions for each strain still requires solid scientific/clinical justification in accordance with the Regulation on Health Claims, particularly for claims related to gastrointestinal or immunomodulatory benefits (EFSA BIOHAZ Panel 2023).

In addition to the above, in the United States, regulations depend specifically on the intended use, that is, food versus supplements. Notifications have been issued for foods that do not pose a risk when used correctly. For example, the United States has recently issued specific notifications for the DSM 17938 strain, supporting its use in various food categories under defined conditions (FDA 2008). However, despite existing regulations, encapsulating these strains introduces an additional layer of regulatory and safety considerations beyond the microorganism itself. Specifically, food quality can be affected by wall materials, as incorporating polymeric matrices based on alginate, chitosan, milk proteins, or plant polymers such as pectins can alter labeling requirements, stability during processing, sensory properties, and consumer acceptance (Cook et al. 2013).

Current technological research focuses on multilayer capsules and designs that enhance cell protection during processing and gastrointestinal stress. Encapsulation strategies that combine *L reuteri* with prebiotic substrates are also being explored to improve viability and functional performance after release.

## 4 Conclusion

The evidence compiled in this review highlights *L. reuteri* as a versatile and highly promising probiotic, whose functional properties extend from antimicrobial and anti‐inflammatory activity to metabolic regulation, antioxidant effects, and applications in gastrointestinal, oral, and systemic health. Until now, the ability of this microorganism to exert benefits has been strongly conditioned by its survival processing, storage and gastrointestinal transit. Encapsulation has emerged as one of the most effective strategies to address these challenges, with ionic gelation, extrusion, spray drying, and some other approaches. According to the literature consulted in this review, alginate remains as the most widely used biopolymer used in encapsulation due to its high biocompatibility and low cost. However, recent studies have demonstrated that some other materials including proteins and polysaccharides improve EE and controlled release. Despite all the progress in this matter, critical gaps remain in translating the laboratory research to industry scale. Most current studies are limited to in vitro models, with fewer investigations exploring performance in food matrices or under real industry‐scale conditions. Moreover, there is a limited knowledge of how encapsulation affects consumer acceptance and regulatory approval pathways. To move forward, future research must prioritize integrated approaches that combine material science, microbiology and clinical validation. Also, multidisciplinary efforts will allow the development of encapsulation platforms with capacity to maintain probiotic viability and ensure functional efficacy in vivo.

Despite the wide variety of functional properties exhibited by *L. reuteri* strains ranging from antimicrobial activity to therapeutic potential studies on their encapsulation remain limited. This limitation makes it difficult to predict their technological behavior and response to various processes. Hence, the importance of expanding research, especially under conditions that replicate real‐life food matrices and industrial processing scenarios.

In the next stage of research, work on encapsulation should move past basic comparisons of insufficient materials and methods. It is essential to consider how these strategies impact release kinetics and sensory response in both food and pharmaceutical applications. Such an approach will support the creation of delivery systems specifically designed to maximize the functional benefits of *L. reuteri* and to demonstrate consistent performance in real‐world applications.

## Nomenclature

ABTS: 2,2‐azino‐bis‐3‐ethylbenzothiazoline‐6‐sulfonic acid
CFU: colony forming units
CAGR: compound annual growth rate CAGR
DPC: dairy products collection
CP: *Cyclocarya paliurus* leaf extracts
DPPH: 2,2‐diphenyl‐1‐picrylhydrazyl
DSM: Deutsche Sammlung von Mikroorganismen
EHDA: electrohydrodynamic atomization
EFSA: European Food Safety Authority
FAO: Food and Agriculture Organization
GITS: gastrointestinal simulation
GRAS: generally recognized as safe
HPI: hemp seed protein isolate
LAB: lactic acid bacteria
LR: *Limosilactobacillus reuteri*
pH: potential of hydrogen
QPS: Qualified Presumption of Safety
SEM: scanning electron microscopy
WHO: World Health Organization

## Author Contributions

**León‐Espinosa Erika Berenice**: conceptualization, methodology, writing – review and editing, investigation, writing – original draft. **Barrios‐Francisco Rigoberto**: methodology, writing – review and editing. **Colin‐Molina Abraham**: investigation, resources, writing – review and editing. **Martínez‐Palma Nikte Yoliztli**: investigation, writing – review and editing. **Rentería‐Ortega Minerva**: conceptualization, methodology, resources, writing – original draft, writing – review and editing, supervision, project administration.

## Conflicts of Interest

The authors declare no conflicts of interest.

## Data Availability

The original contributions presented in the study are included in the article, further inquiries can be directed to the corresponding author.
